# Association between vasectomy and risk of testicular cancer: A systematic review and meta-analysis

**DOI:** 10.1371/journal.pone.0194606

**Published:** 2018-03-22

**Authors:** Haifeng Duan, Tuo Deng, Yiwen Chen, Zhijian Zhao, Yaoan Wen, Yeda Chen, Xiaohang Li, Guohua Zeng

**Affiliations:** 1 Department of Urology, Minimally Invasive Surgery Center, The First Affiliated Hospital of Guangzhou Medical University, Guangzhou, Guangdong, China; 2 Guangdong Key Laboratory of Urology, Guangzhou, Guangdong, China; University of Zurich, SWITZERLAND

## Abstract

**Objectives:**

A number of researchers have reported that vasectomy is a risk factor for testicular cancer. However, this conclusion is inconsistent with a number of other published articles. Hence, we conducted this meta-analysis to assess whether vasectomy increases the risk of testicular cancer.

**Materials and methods:**

We identified all related studies by searching the PubMed, Embase, and Cochrane Library database from January 01, 1980 to June 01, 2017. The Newcastle-Ottawa Scale (NOS) checklist was used to assess all included non-randomized studies. Summarized odds ratios (ORs) and 95% confidence intervals (CIs) were used to assess the difference in outcomes between case and control groups. Subgroup analyses were performed according to the study design and country.

**Results:**

A total of eight studies (2176 testicular cancer patients) were included in this systematic review and meta-analysis. Six articles were case-control studies, and two were cohort studies. The pooled estimate of the OR was 1.10 (95% CI: 0.93–1.30) based on the eight studies in a fixed effects model. Two subgroup analyses were performed according to the study design and country. The results were consistent with the overall findings. Publication bias was detected by Begg’s test and Egger’s test and *p* values > 0.05, respectively.

**Conclusions:**

Our meta-analysis suggested that there was no association between vasectomy and the development of testicular cancer. More high-quality studies are warranted to further explore the association between vasectomy and risk of testicular cancer.

## Introduction

Testicular cancer accounts for 1% of male tumors[1]. This cancer is the most common cancer for men aged 20 to 39 years[2]. Testicular cancer’s incidence and mortality rate have been gradually increasing. Testicular cancer caused 8300 deaths worldwide in 2003 and 7000 deaths in 1990, an increase of 19%[3]. Ghazarian et al[4] showed that the incidence of testicular cancer continues to increase in the United States. Vasectomy is a simple surgical procedure that can be performed with local lidocaine anesthesia in the outpatient. Vasectomy is the most effective permanent form of contraception available to men. It is reported that the prevalence of vasectomy was approximately 8% in China[5]. New Zealand, Canada and the United Kingdom have a higher prevalence of vasectomy: 25% of the married men and 57% in the age group of 40–49[6,7].

However, several studies showed that vasectomy was associated with testicular cancer[8,9] and prostate cancer[10,11]. This association especially caught the public’s attention because of the extensive use of vasectomy. Recently, Liu et al[12] published a meta-analysis showing that vasectomy may not contribute to the risk of prostate cancer. The relation between vasectomy and testicular cancer has not been thoroughly elucidated to date. Several studies have reported a positive association between vasectomy and testicular cancer[8,9]. Other studies found the opposite results[13,14,15,16,17,18,19]. However, the number of patients in these studies are small, and the level of evidence is not high.

Therefore, we conducted this systematic review and meta-analysis to assess the association between vasectomy and risk of testicular cancer, aiming to provide further evidence and guidelines for the general public.

## Materials and methods

### Search strategy

A comprehensive electronic literature search of Pubmed, Embase, and Cochrane Library databases was conducted by two members to identify studies which assessed the association between vasectomy and the risk of testicular cancer. Separate searches were performed with the following search terms: (‘vasectomy’ or ‘deferentectomy’ or ‘vasoligation’ or ‘vasoligature’) AND (‘testicular neoplasms’ or ‘testicular cancer’). The detailed retrieval process is shown in S2 File. References of all included studies were also checked for potential papers. This search was repeated until no additional articles were found. The full date range for the search is from January 01, 1980 to June 01, 2017.

### Study selection, inclusion and exclusion criteria

We performed an initial screening based on the titles and abstracts. If uncertain, a subsequent full-text assessment was conducted. The second screening was a full-text review. Populations for this review and meta-analysis were broadly inclusive, involving any country and race. Studies were included if they met the following criteria: (i) the study design was a cohort, case-control study or randomized controlled trial; (ii) contained vasectomy and testicular cancer measures; (iii) studies that reported the odds ratio (OR) or relative risk (RR) relating vasectomy to testicular cancer outcome and the corresponding 95% confidence interval (CI) (or sufficient data to calculate them); and (iv) the publication language was confined to English. Exclusion criteria were (i) duplicate literature; (ii) reviews, meeting summaries, and editorials comments; and (iii) studies that had no control group.

### Study quality assessment and data extraction

Two researchers (Duan and Chen) independently extracted data from all eligible studies. Any differences were discussed or decided by the third reviewers (Deng). Data extraction was performed using a standardized data collection form. The following data were collected from each of the selected articles: the name of the first author, data source, study type, age, study period, publication year, length of follow-up, the number of patients and participants, OR, RR, 95% CIs and statistical adjustments for confounding factors.

The Newcastle-Ottawa Scale (NOS) was applied to assess the quality of all studies. The NOS checklist contains three parameters of quality: (i) selected population, (ii) comparability of groups, and (iii) assessment of either the exposure or outcome of interest for case-control or cohort studies. Each study was assigned a score of 0–9. The studies scored greater than or equal to 7 were considered to be high quality articles.

### Statistics analysis

In the meta-analysis, RevMan analytical software package (Version 5.3, Cochrane Collaboration, Oxford, UK) was used to combine the extracted data. The pooled OR or RR and the corresponding 95% CIs were calculated to assess the relationship between vasectomy and risk of testicular cancer. Heterogeneity was assessed using the chi-square test based *Q*- and *I*^2^- statistic. If heterogeneity was not present (*P* > 0.10, *I*^2^ < 50%), a fixed-effect model was used to calculate the combined OR values. Otherwise, a random-effect model was used. Subgroup analysis was performed according to the study design and country. All results in this analysis were considered as significant only with a two-tailed *P* < 0.05. Both the Begg’s test and the Egger’s test were performed using Stata 12.1 (Stata Corp., College Station, TX) to determine whether publication bias existed.

## Results

**Fig 1:**
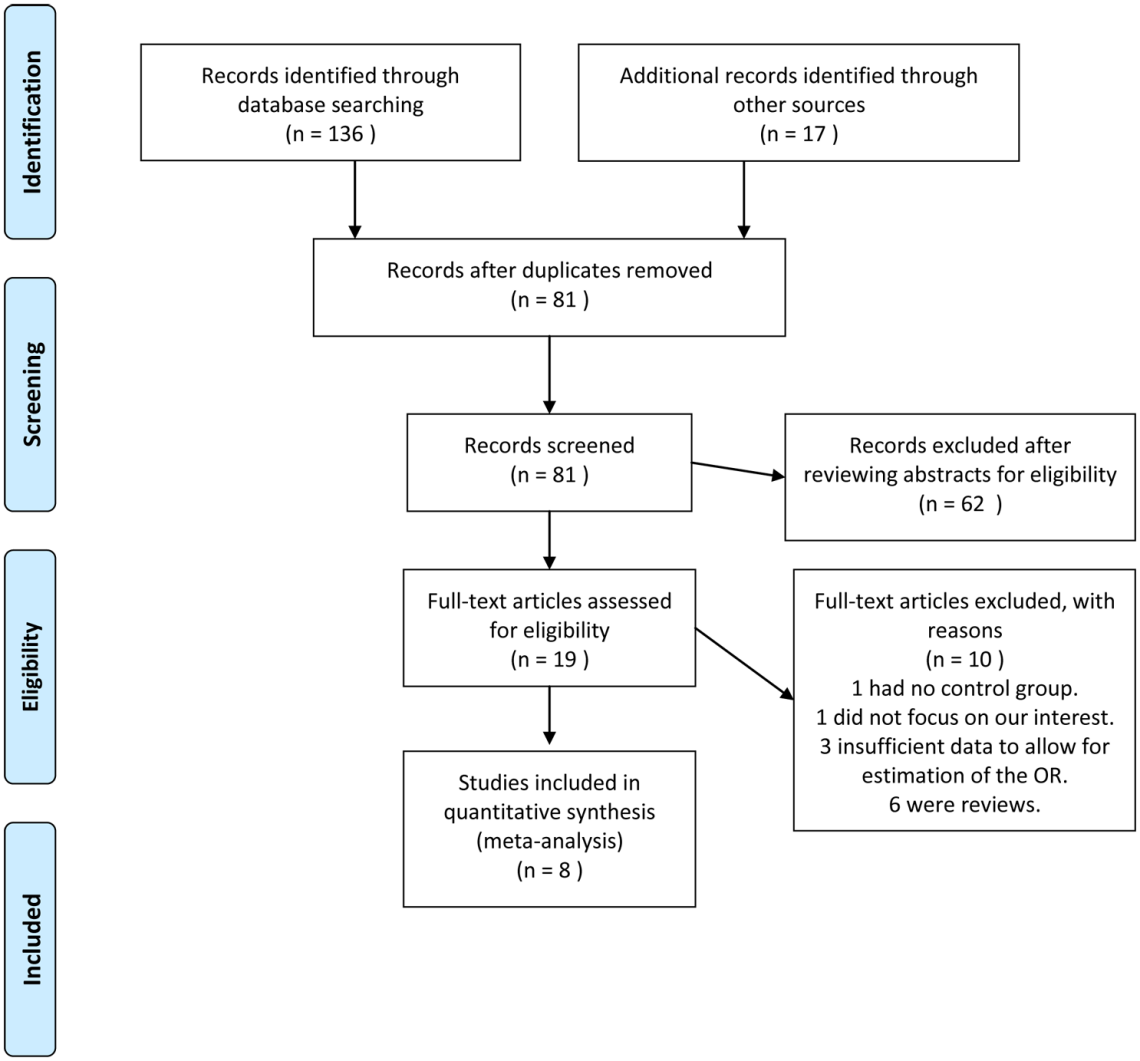
Flow diagram of studies selection process.

After screening the manuscripts, we included eight articles (Strader et at., 1988; Forman et at., 1994; Moss et al., 1986; Brown et at., 1987; Rosenberg et at., 1994; Swerdlow et at., 1987; Nienhuis et at., 1992; Eisenberg et at., 2015). The detailed retrieval process is shown in Fig 1. The characteristics of qualified studies are listed in Table 1. In total, 11,141 participants in case-control studies and 908,927 in cohort studies (a total of 2176 testicular cancer patients were included in this systematic review and meta-analysis. Six articles were case-control studies, and two were cohort studies. Five studies were conducted in the United States of America (USA), and three were in England.

**Table 1:** The characteristics of qualified researches.

**First author**	**Publication time**	**Country**	**Study design**	**Age (years)**	**Number of cases**	**Number of controls**	**OR**	**95%CI**	**Diagnosis**	**Variable adjustment**	**Quality scores**
D Forman[13]	1994	England	Case-control	15–49	794	794	1.1	0.8–1.5	testicular germ cell tumor	Same practitioner	7
Moss AR [14]	1985	Americans	Case-control	>18	173	212	0.6	0.3–1.2	testicular germ cell tumor	Friends, race, age	8
Brown LM [15]	1987	American	Case-control	18–42	266	254	1	0.3–3.3	testicular cancer	same hospital, other malignancy, age, race, vital status.	7
Rosenberg L [16]	1994	American	Case-control	<70	132	7027	0.8	0.4–1.9	testicular cancer	no history of cancer	7
Strader CH [8]	1987	American	Case-control	20–69	228	513	1.5	1.0–2.2	testicular germ cell tumor	Residence time, education, religion.	7
Swerdlow AJ [17]	1986	England	Case-control	>10	259	489	1.1	0.63–2.04	testicular cancer	town, age	7
First author	Publication time	Country	Study design	Age (years)	Number of exposed groups	Number of non exposed groups	RR	95%CI	Diagnosis	Variable adjustment	Quality scores
Nienhuis H [18]	1992	England	Cohort study	25–49	13246	22196	0.46	0.1–1.4	testicular cancer	age	6
Eisenberg ML [19]	2014	American	Cohort study	18–50	112655	760830	1.27	0.94–1.73	testicular cancer	age	5

As shown in Table 2, according to the NOS checklist, six studies with scores ≥ 7 stars were considered high quality, and the remaining two studies were medium quality for 5 and 6 stars, respectively.

**Table 2:** The Newcastle-Ottawa Scale (NOS).

**First author**	**Quality evaluation**	**Case definition**	**Representativeness**	**Selection of Controls**	**Definition of Controls**	**Comparability**	**Ascertainment of exposure**	**Same method?**	**Non-Response rate**
D Forman[13]	7	1	1	1	1	1	1	1	0
Moss AR [14]	8	1	1	1	1	1	1	1	1
Brown LM [15]	7	1	1	0	1	1	1	1	1
Rosenberg L [16]	7	1	1	0	1	1	1	1	1
Strader CH [8]	7	1	1	1	1	1	1	1	0
Swerdlow AJ [17]	7	1	1	0	1	1	1	1	0
First author	Quality evaluation	Representativeness of exposed cohort	Selection ofnon exposed cohort	Ascertainment of exposure	outcome not present before study	Comparability	Assessment of outcome	follow-up long enough	Non-Response rate
Nienhuis H [18]	6	1	1	1	1	1	1	0	0
Eisenberg ML [19]	5	1	0	1	1	1	1	0	0

## Outcomes

**Fig 2:**
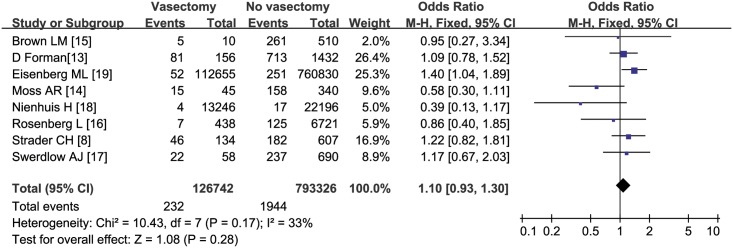
Forest plot of all included studies.

Overall, the pooled estimate of the OR was 1.10 (95% CI: 0.93–1.30, *P* = 0.28) based on eight studies in a fixed effects model with no significant heterogeneity (*I*^*2*^ = 33%, *P* = 0.15) (Fig 2). One of our subgroup analyses was carried out according to research types.

**Fig 3:**
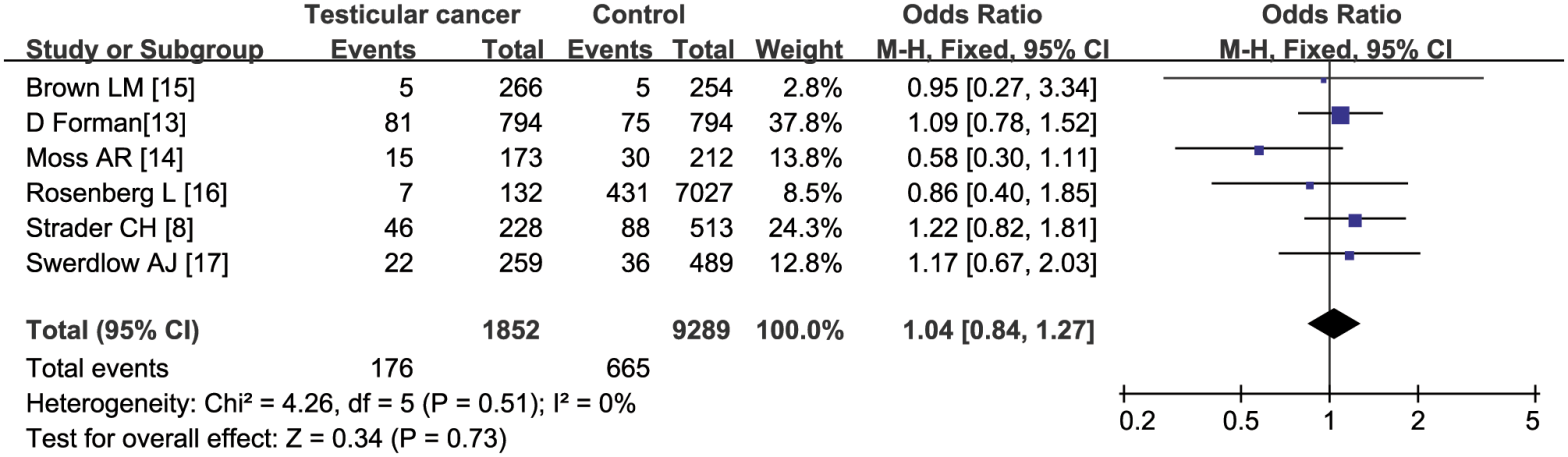
Subgroup analysis of case-control studies.

**Fig 4:**
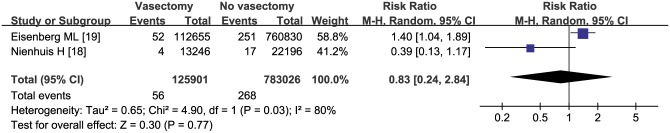
Subgroup analysis of cohort studies.

The pooled estimate of the OR was 1.04 (95% CI: 0.84–1.27, *P* = 0.69) based on six case-control studies in a fixed effects model, without heterogeneity (*I*^*2*^ = 0%, *P* = 0.51) (Fig 3). The pooled estimate of the OR was 0.83 (95% CI: 0.24–2.84, P = 0.77) based on two cohort studies in a random effects model with significant heterogeneity (*I*^*2*^ = 80%, *P* = 0.03) (Fig 4)

**Fig 5:**
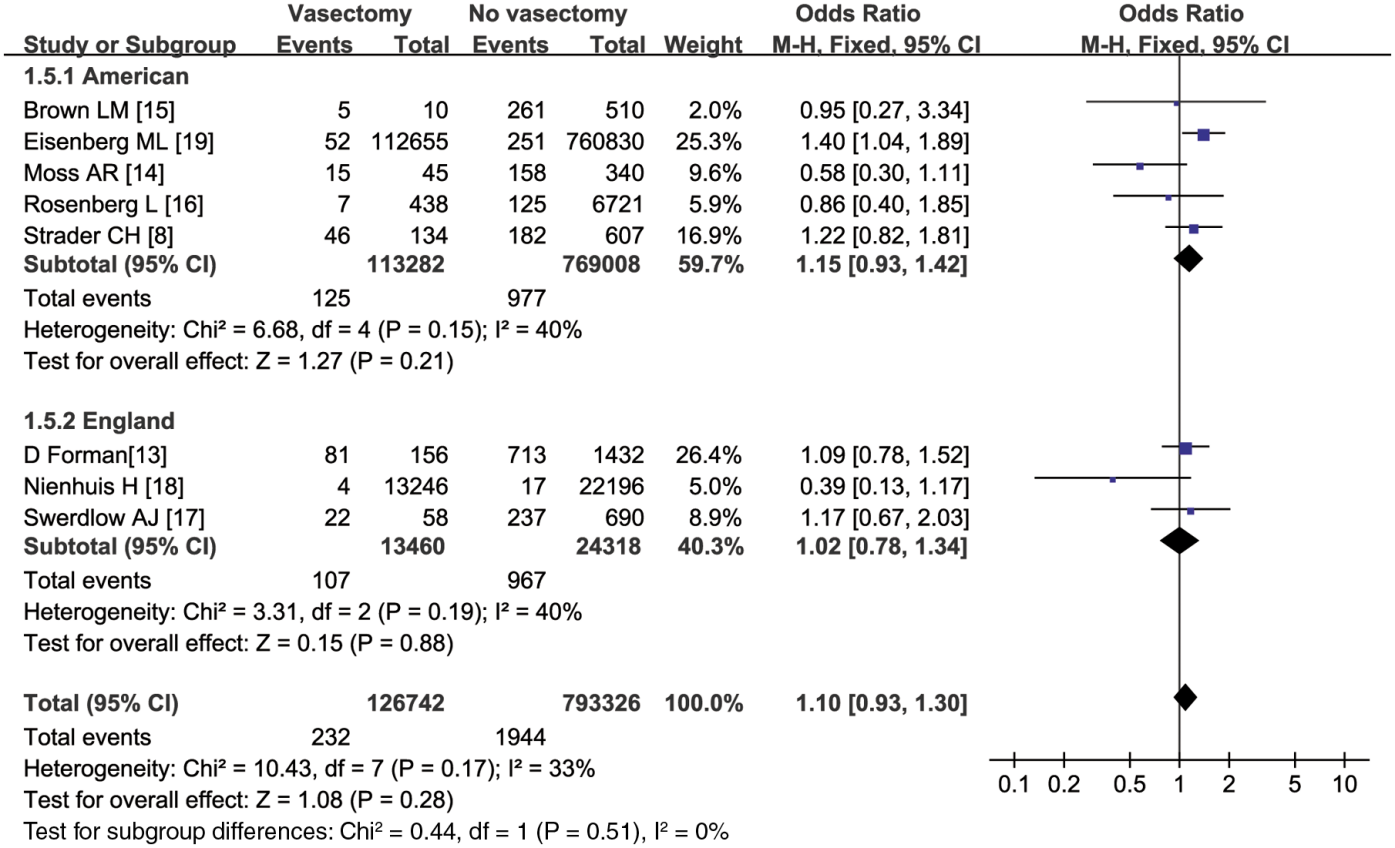
Subgroup analysis based on countries.

The other subgroup analysis was carried out based on countries. The pooled estimate of the OR was 1.15 (95% CI: 0.93–1.42, *P* = 0.21), based on five USA studies in a fixed effects model without significant heterogeneity (*I*^*2*^ = 40%, *P* = 0.151) (Fig 5). The pooled estimate of the OR was 1.02 (95% CI: 0.78–1.34, *P* = 0.88), based on three English studies in a fixed effects model with mild heterogeneity (*I*^*2*^ = 40%, *P* = 0.19) (Fig 5).

## Publication bias

**Fig 6:**
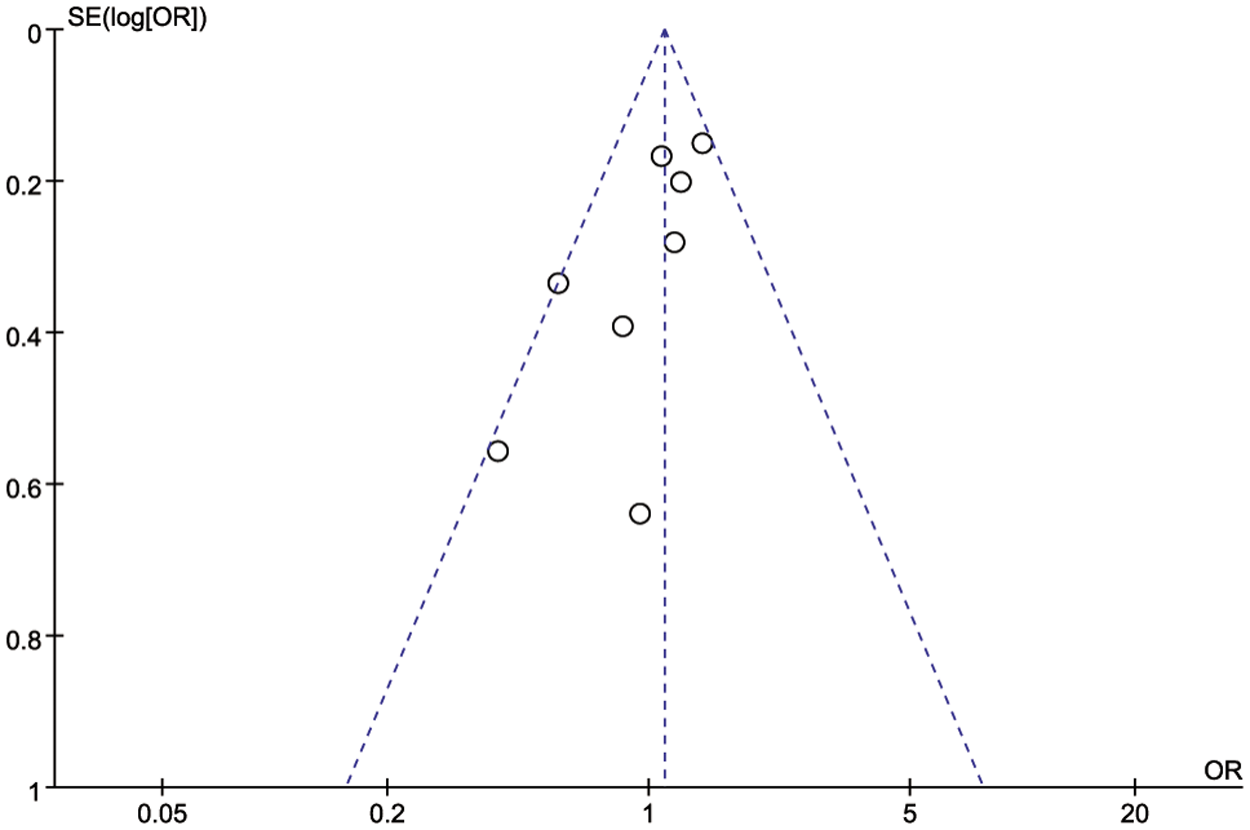
Funnel plots.

Both the Begg’s test and Egger’s test were conducted to assess publication bias, indicating no significant publication bias among all the included studies (Fig 6).

## Discussion

1. To the best of our knowledge, this report presents the first systematic review and meta-analysis assessing the association between vasectomy and the risk of testicular cancer. A total of 11,141 participants in case-control studies and 908,927 in cohort studies (a total of 2176 testicular cancer patients) were included in our systematic review and meta-analysis. Our results showed that there was no significant association between vasectomy and the development of testicular cancer. Vasectomy, as a common method of permanent birth control, should not be forbidden unless there are more high quality papers reporting the positive association between vasectomy and testicular cancer. More studies are required to further explore the association between vasectomy and risk of testicular cancer.

2. There were two studies reporting positive association between vasectomy and testicular cancer[8,9]. Strader et al[8] reported that OR was 1.5 and 95 CI was 1.0–2.2. The authors only found this association in Catholic men. A history of vasectomy was reported with approximately equal frequency by Catholics and non-Catholics in the case group. However, a great difference was reported by Catholics and non-Catholicsin the control group, 6.3% VS 19.7%. The authors explained that was mainly owing to selective underreporting by Catholic men. In this study, there had been a extended period time between vasectomy and the study. Information bias should be controlled by querying physicians and checking the medical records, not just based on the patient;’s self—report. Cale et al[9] suggested that vasectomy could accelerate the development of testicular cancer. However, it has limited cases(37 testicular cancer patients), no well-controlled group and it remains unclear whether testicular cancer existed before vasectomy. So it could not provide good evidence. A well-designed research is required to further explore whether vasectomy accelerates the development of testicular cancer. The largest of these case-control studies (794 testicular cancer patients) were conducted by face-to-face interviewing and showed no association between vasectomy and risk of testicular cancer[13]. The median time between case diagnosis and interview was only 10 months, and patients’ self-reported medical histories were confirmed by general practitioners. That could well reduce the information bias, giving more convincing results. To get more reliable results, two cohort studies were performed[18,19]. The first was a retrospective cohort study[18], performed in 1992. A total of 13,246 men undergoing vasectomy and 22,196 comparison subjects were included, showing there was no evidence of an increase associated with vasectomy in the incidence of testicular cancer. The information was recorded according to medical records. The research should record the diseases of cryptorchidism, infertility and testicular injury, which increase the risk of testicular cancer. The other cohort study, which analyzed US claims data, was performed in 2014[19] and was the most recent of the included studies. A total of 112,655 vasectomized men and 760,830 control men were included. Few or no infertile men were included in the vasectomized men group. The research showed that vasectomy was not a risk factor for testicular cancer. In our opinion, we cannot conclude that vasectomy increased risk of testicular cancer based on the current literature.

3. Several human immune system effects caused by vasectomy were reported in a number of studies. For example, 50% of the men with vasectomy were found to have circulating spermatozoal antibodies[20]. The level of testosterone was confirmed to be unchanged after vasectomy[21]. However, all studies to date have lacked information on the mechanism by which vasectomy increases the risk of testicular and prostate cancer. The World Health Organization (WHO) meeting in 1991 concluded that there was no biological mechanism to account for any association between vasectomy and prostate cancer.

4. Potential bias was unavoidable. First, men who had a vasectomy were more proactive in seeking medical care if they sensed an abnormality in their bodies[22]. Thus, the detection of testicular cancer would be more possible for men with a vasectomy surgery compared with men who have no vasectomy. Second, cryptorchidism, infertility, exposure to organochlorine pesticides and some unreported factors could increase the risk of testicular cancer[15,23]. However, it is impossible to ensure that the risk factors for testicular cancer are evenly distributed to two groups. To reduce the bias, subgroup analysis was conducted according to research types. Neither case-control group nor cohort-study group showed positive association between vasectomy and the risk of testicular cancer. Third, the development of the testicular cancer may take a long time. The follow-up time for each study was different. If the follow-up time was not long enough, it would produce false negative results.

5. Our meta-analysis had several potential limitations. First, only British and American articles were included. This would limit the results of our research to other populations. Second, due to the limited data available in the original article, we could not perform more comprehensive and detailed subgroup analysis, such as subgroups based on the staging of testicular cancer, follow-up time, and age. Third, most of the included articles were not recent. More newly published studies are required.

## Conclusions

Our meta-analysis suggested that there is no association between vasectomy and the development of testicular cancer. More studies are required to further explore the association between vasectomy and the risk of testicular cancer.

## Supporting information

S1 File PRISMA checklist. (DOC)

S2 File Detailed search criteria in PubMed. (DOCX)

S1 Table The 19 references which were excluded by reading the full texts. (XLSX)
