# Supplementary material for: Association between vasectomy and risk of testicular cancer: A systematic review and meta-analysis
Source: PLoS One. 2018 Mar 22;13(3):e0194606. doi: 10.1371/journal.pone.0194606 (PMC5864054; doi:10.1371/journal.pone.0194606)
Supplement: S2 File — (DOCX) [file pone.0194606.s002.docx]

**Detailed search strategy for PUMED database**

#1 vasectomy[MeSH Terms]

#2 vasectomy[All Fields]

#3 deferentectomy[All Fields]

#4 vasoligation[All Fields]

#5 vasoligature[All Fields]

#6 #1 OR #2 OR #3 OR #4 OR #5

#7 testicular neoplasms[MeSH Terms]

#8 testicular[All Fields] AND neoplasms[All Fields]

#9 testicular neoplasms[All Fields]

#10 testicular[All Fields] AND cancer[All Fields]

#11 testicular cancer[All Fields]

#12 #7 OR #8 OR #9 OR #10 OR #11

#13 1980/1/1[Date - Publication] : 2017/6/1[Date - Publication]

#14 #6 AND #12 AND #13

**(((((((vasectomy[MeSH Terms]) OR vasectomy) OR deferentectomy) OR vasoligation) OR vasoligature)) AND (((((testicular neoplasms[MeSH Terms]) OR (testicular AND neoplasms)) OR testicular neoplasms) OR (testicular AND cancer)) OR testicular cancer)) AND ("1980/1/1"[Date - Publication] : "2017/6/1"[Date - Publication])**
